# Development of GMDR-GPU for Gene-Gene Interaction Analysis and Its Application to WTCCC GWAS Data for Type 2 Diabetes

**DOI:** 10.1371/journal.pone.0061943

**Published:** 2013-04-23

**Authors:** Zhixiang Zhu, Xiaoran Tong, Zhihong Zhu, Meimei Liang, Wenyan Cui, Kunkai Su, Ming D. Li, Jun Zhu

**Affiliations:** 1 Institute of Bioinformatics, Zhejiang University, Hangzhou, China; 2 State Key Laboratory for Diagnosis and Treatment of Infection Diseases, First Affiliated Hospital, Zhejiang University School of Medicine, Hangzhou, China; 3 Department of Psychiatry and Neurobehavioral Sciences, University of Virginia, Charlottesville, Virginia, United States of America; Yale University, United States of America

## Abstract

Although genome-wide association studies (GWAS) have identified a significant number of single-nucleotide polymorphisms (SNPs) associated with many complex human traits, the susceptibility loci identified so far can explain only a small fraction of the genetic risk. Among other possible explanations, the lack of a comprehensive examination of gene–gene interaction (G×G) is often considered a source of the missing heritability. Previously, we reported a model-free Generalized Multifactor Dimensionality Reduction (GMDR) approach for detecting G×G in both dichotomous and quantitative phenotypes. However, the computational burden and less efficient implementation of the original programs make them impossible to use for GWAS. In this study, we developed a graphics processing unit (GPU)-based GMDR program (named GWAS-GPU), which is able not only to analyze GWAS data but also to run much faster than the earlier version of the GMDR program. As a demonstration of the program, we used the GMDR-GPU software to analyze a publicly available GWAS dataset on type 2 diabetes (T2D) from the Wellcome Trust Case Control Consortium. Through an exhaustive search of pair-wise interactions and a selected search of three- to five-way interactions conditioned on significant pair-wise results, we identified 24 core SNPs in six genes *(FTO:* rs9939973, rs9940128, rs9922047, rs1121980, rs9939609, rs9930506; *TSPAN8:* rs1495377; *TCF7L2:* rs4074720, rs7901695, rs4506565, rs4132670, rs10787472, rs11196205, rs10885409, rs11196208; *L3MBTL3:* rs10485400, rs4897366; *CELF4:* rs2852373, rs608489; *RUNX1:* rs445984, rs1040328, rs990074, rs2223046, rs2834970) that appear to be important for T2D. Of these core SNPs, 11 in *FTO*, *TSPAN8*, and *TCF7L2* have been reported to be associated with T2D, obesity, or both, providing an independent replication of previously reported SNPs. Importantly, we identified three new susceptibility genes; i.e., *L3MBTL3, CELF4,* and *RUNX1,* for T2D, a finding that warrants further investigation with independent samples.

## Introduction

During the past several years, searching susceptibility loci for various human diseases has been revolutionized by genome-wide association studies (GWAS). Although a significant number of single-nucleotide polymorphism (SNP) have been reported to be associated with various human complex traits [1], only a small fraction of the genetic risk can be explained by those identified SNPs for each trait, often termed the “missing heritability” problem [2], [3]. Although many factors such as rare genetic variation, structural variation, epigenetics, gene–environmental interactions may have contributed to this missing heritability [1]–[4], gene–gene interaction (G×G) is thought to be an important component of multifactorial disease genetics because of the complexity of biological systems [5], [6]. However, examination of G×G in GWAS is often limited by the lack of a large sample, inadequate statistical methods, and unavailability of appropriate software and computational capacity [5]–[7].

To deal with the challenge of detecting G×G, much research is under way on improving both statistical and computational methodologies. A number of statistical methods and corresponding software packages have been developed, which range from simple exhaustive searches to data-mining and machine-learning approaches to Bayesian model selection [6]. On the basis of computational speed, and presumably ease of use, it was implied by Cordell [6] that the programs PLINK [8], Random Jungle [9], and BEAM [10] are the most computationally feasible methods for detecting G×G in genome-wide data.

Regarding the multifactor dimensionality reduction (MDR) method [11]–[13] or its improvements such as entropy-based interpretation methods [14], the use of odds ratios [15], log-linear methods [16], generalized linear models [17], and permutation testing [18], one of the major concerns is that these programs are incapable of scale-up for analyzing GWAS data, as they were not designed with genome-wide data in mind and thus could fail owing to memory and disk usage issues [6]. However, even though the MDR and its extensions are incapable of handling GWAS data, they have been applied to a wide range of genetic association studies where only a small number of SNPs were examined for each sample [19]. For example, Andrew and colleagues used MDR to model the relation between SNPs in DNA repair enzyme genes and susceptibility to bladder cancer [20]. The GMDR has been successful in identifying the significant interaction of *CHRNA4* with *CHRNB2* [21], *NTRK2* with *BDNF* [21], and *GABBR1* with *GABBR2* [22] in nicotine dependence, of *LEPR* and *ADRB2* in obesity [23], and of *HNF4A* and *KCNJ11* in type 2 diabetes (T2D) [24]. However, because most of these findings have not been confirmed in independent studies, they should be interpreted with caution.

Although two general strategies, the filter approach and the stochastic search algorithm, have been proposed for scaling up the capability of MDR for analyzing GWAS data [19], neither addresses the issue related to the MDR algorithm *per se,* which is computational intensiveness and infeasibility in the original Java implementation of the algorithm. Thus, the primary objective of this study was to develop an effective software (i.e., GMDR-GPU) that can run much more effectively in a more sophisticated computing system. As a demonstration of this newly developed GMDR-GPU program, we used it to analyze the type 2 diabetes (T2D) phenotype from the Wellcome Trust Case Control Consortium (WTCCC) study [25] with the goals not only of verifying susceptibility loci but also of identifying novel ones for this disease.

## Materials and Methods

### Description of GMDR-GPU Software

**Figure 1:**
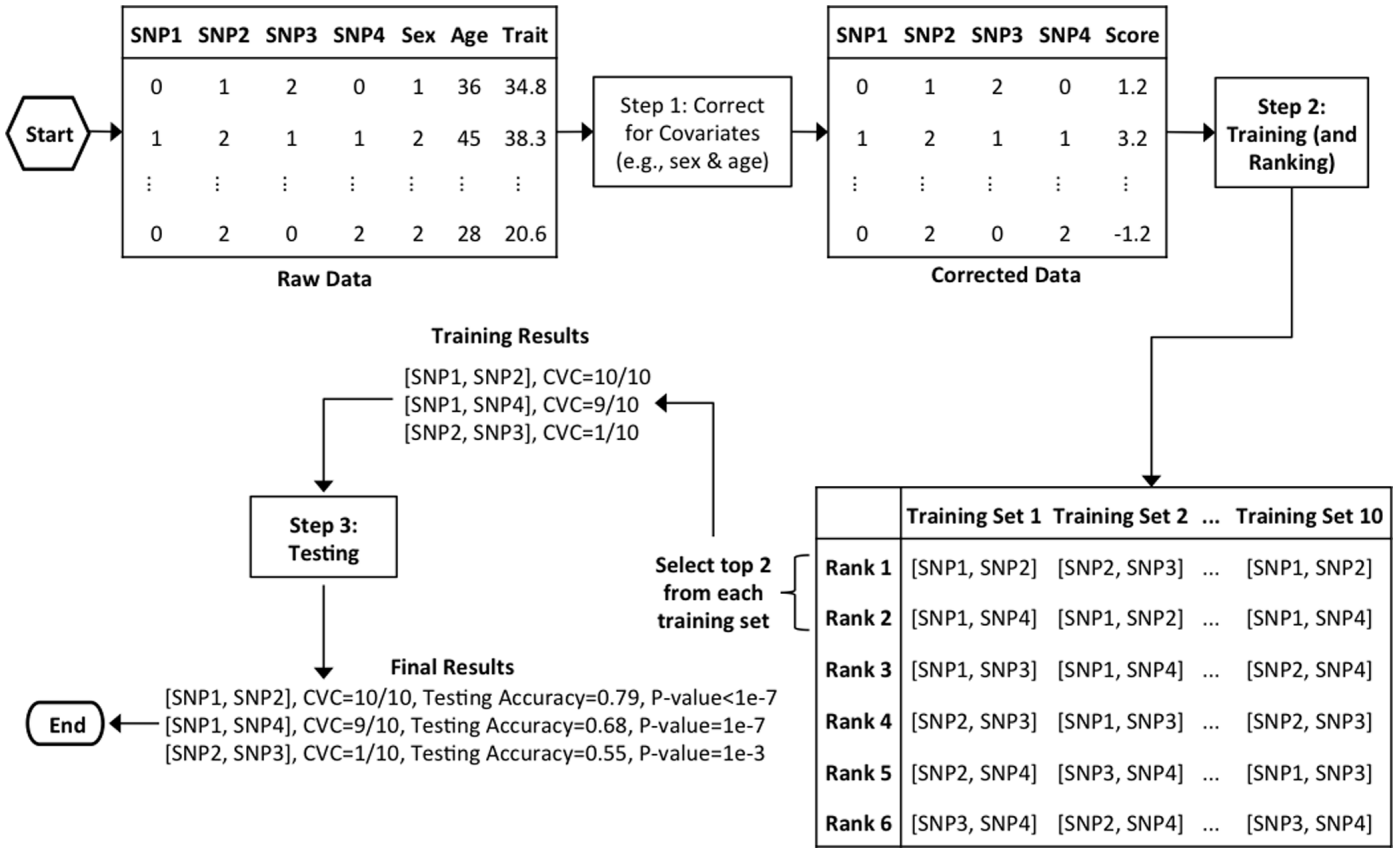
Working process of the GMDR-GPU program for conducting a two-dimensional interaction search on a sample consisting of four SNPs, two covariates, and a quantitative phenotype.

The GMDR-GPU software implements GMDR using standard C++ and CUDA 4.0 to make use of multiple graphics processing units (GPUs). The source code is cross-platform and can be built for Windows, Linux, or Mac OS X. As illustrated in Figure 1 for data consisting of four SNPs, two covariates, and a continuous phenotype, the analysis process of GMDR-GPU can be summarized as three main steps.

#### Step 1: Justification for covariates

Compared with other MDR algorithms, one of the major advantages of GMDR [17] is the allowance of covariate correction, which takes place in this step. By taking any covariate and the phenotypic data as input, GMDR-GPU calculates a “*score”* statistic for each subject based on a generalized linear model under different distributions; i.e., normal, Poisson, and Bernoulli [17]. For the sake of user friendliness, GMDR-GPU assumes that binary traits follow a Bernoulli distribution, so the scores are actually residuals of logistic regression relating the phenotype to the covariates, whereas for quantitative traits, GMDR-GPU assumes they follow a normal distribution, so the scores are actually residuals of linear regression. With this approach, users do not need to worry about which regression model should be used for justification of covariates. However, advanced users have the option of providing the scores directly to the program so they can use their own regression models to calculate the scores and then use GMDR-GPU to complete the remaining computation, which requires deliberate optimization of the program for handling the intensive computational burden. In the example shown in Figure 1, the data contain two covariates, sex and age, whereas the phenotype is continuous, so GMDR-GPU calculates the score of each subject on the basis of a linear regression.

#### Step 2: Training and ranking all the SNP combinations included in the data following a cross-validation framework

After appropriate justification of covariates, GMDR-GPU performs intensive computation with the goal of selecting those SNP combinations showing the strongest association signals as candidate G×G models. This is realized by a training step based on a cross-validation framework [11], [17]. The data are randomly divided into *K* partitions of equal size for *K*-fold cross-validation, where *K* is a default to 10. Accordingly, *K* training sets are formed where each set consists of all but one of the *K* data partitions. Within each training set, the genotypes of all the SNP combinations are classified as high-risk or low-risk cells according to the genotype and score data; i.e., the justified phenotypic data; and all the SNP combinations are ranked by their training (classification) accuracies. Those combinations with the highest training accuracies are then selected as the input for *step* 3 to complete further testing. With the default option, in each training set, only the SNP combination with a rank of 1 is selected. However, users have the option of selecting multiple SNP combinations with the highest training accuracies from each training set. In the example provided in Figure 1, the data contain four SNPs, so there are 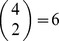 SNP combinations for a two-dimensional interaction search. In each of the 10 training sets, the top two SNP combinations with the highest training accuracies are selected (the values of the training accuracies are not depicted in Figure 1).

This step also outputs the cross-validation consistency (CVC) statistics for the selected SNP combinations to indicate their accuracies as predictive SNP–SNP interaction models. The CVC statistic of an SNP combination is defined by the number of times it is selected from all the training sets. The higher the CVC, the more robust the SNP combination as a predictive interaction model. By default, in this step, only those SNP combinations with CVCs>(*K*/2)/*K* are outputted as possible G×G models. This step is the one in which the massively parallel computation technique of GPU and program performance optimization begin to be applied. The GMDR-GPU generates the SNP combinations in the CPU to take advantage of its fast sequential execution feature, while at the same time, it distributes the calculation of all the training accuracies for an SNP combination to one of the thousands of GPU threads using a round-robin scheduling approach [26]. This tool takes advantage of the many-core architecture of a GPU so that a large number of SNP combinations can be trained simultaneously. Each GPU thread contains a selector to pick the combinations with the highest training accuracies from those combinations distributed to the thread and sends the selected combinations to the central processing unit (CPU). The CPU does the final selection and outputs those SNP combinations having the highest training accuracies and their CVCs as the training result.

During the GPU computation, GMDR-GPU utilizes global memory, constant memory, and registers of the GPU memory architecture to achieve optimized memory consumption with respect to space and access speed. Because the amount of GPU memory is usually limited, the genotype data are stored in global memory in a compact way. The scores calculated by *step* 1 are stored in constant memory to accelerate access. The current version of GWAS-GNDR can analyze a maximum of 10,000 subjects for each run.

#### Step 3: Determining the testing accuracies and significance of the SNP combinations selected by Step 2

After identifying the candidate interaction models in *Step* 2, GMDR-GPU enters the third step to predict how likely it is that those models represent strong association signals from other independent replicate datasets, which is measured by “testing accuracies” and “*P* value”. For each of the *K* training sets formed in *Step* 2, the data partition that is not contained in the training set is taken as the testing set. For each SNP combination selected by *Step* 2, testing accuracy is calculated on the basis of the genotype classification in *Step* 2 in the corresponding training set; and its final testing accuracy output by this step, which we define as “*observed testing accuracy*,” is calculated by averaging its testing accuracies among all the sets.

The significance or *P* value is determined by a permutation test based on the observed testing accuracies. For each SNP combination selected by *step* 2, the scores are permuted, the genotypes in all the training sets are re-classified, and testing accuracy is re-calculated; this procedure is repeated *N* times, where *N* is a power of 10 specified by each user. The *P* value of an interaction model is defined by *M*/*N*, where *M* is the number of times the re-calculated testing accuracy is as high as the observed testing accuracy. The parallel computation technique of GPU also applies here. The permutation and recalculation repetitions are distributed to all the GPU threads in parallel, so that thousands of repetitions can be run simultaneously. Therefore, users can set *N* to a very large number to increase the precision of the *P* value. Because the permutation test is computationally intensive, we set 10^7^ permutations as a default value of the current version of GMDR-GPU program, although all users has a choice of changing it based on their objectives and the computational capacity.

In short, the current version of GMDR-GPU supports cross-validation consistency, testing accuracy, permutation testing, and high-dimensional interaction analysis. It also permits selecting multiple top-listed interaction models based on cross-validation consistency and testing accuracy with the goal of detecting multiple interactions for a given order of interaction model. The program can run on any computer system equipped with CUDA-enabled GPUs and requires a CUDA 4.0 (or later version) driver.

### Description of WTCCC T2D Data Used in this Study as an Application

A detailed description of the WTCCC study sample can be found in the original paper [25]. Briefly, the dataset includes seven major human disorders: types 1 and 2 diabetes, bipolar disease, coronary artery disease, Crohn’s disease, hypertension, and cardiovascular disease. Each disease is represented by about 2,000 individuals and about 3,000 shared controls. The majority of the subjects are of European ancestry. All the individuals were genotyped using Affymetrix GeneChip 500 K arrays. Given that the primary purpose of this communication to report the development of GMDR-GPU, only the T2D phenotype of this GWAS dataset is reported in this paper.

## Results

### Evaluation of Performance of GMDR-GPU

To evaluate the speed of GMDR-GPU relative to the original Java implementation of GMDR, we conducted a series of performance tests for the Java version GMDR on a server equipped with an Intel® Xeon® X5680 CPU (3.33 GHz) and 96 GB of RAM. Six Tesla C2070 GPUs on the same server were used to test the speed of GMDR-GPU. Performance tests were run on a simulated dataset containing 5,000 subjects with a number of SNPs ranging from 10^3^ to 10^6^ per subject (Table 1). We performed an exhaustive two-dimensional search of each simulated sub-dataset by setting all other running parameters to default values. A CPU program of the same GMDR algorithm was also written in C++ to measure the speed-up obtained using the GPU rather than a CPU, which was run on the same CPU with the same amount of RAM using the same dataset.

**Table 1:** Comparison of time required for exhaustive pair-wise search in 5,000 subjects with 10^3^ to 10^6^ SNPs per sample among the 1- and 6-GPU, C++ CPU, and Java CPU implementations of GMDR-GPU.

Implementation	10^3^ SNPs	10^4^ SNPs	10^5^ SNPs	10^6^ SNPs
CPU (Java)	2.7 hr	11 d *	3 yr *	300 yr *
CPU (C++)	24 min	1.7 d	170 d *	48 yr *
1 GPU	2 sec	4.5 min	7.3 hr	31 d *
6 GPUs	<1 sec	45 sec	75 min	5 d

* Estimated from the running time of searches of smaller data sets.

As shown in Table 1, GMDR-GPU running on a 1 Tesla C2070 was about 550 times faster than the single-core C++ CPU version and about 3,500 times faster than the single-core Java CPU version. We also tested the performance of GMDR-GPU with multiple GPUs compared with a single GPU and found that GMDR-GPU running on six GPUs was about six times faster than that running on one GPU. In other words, GMDR-GPU achieves perfect scalability when running on multiple GPUs.

### Application of GMDR-GPU to WTCCC T2D Phenotype

To provide an example of GWAS data analysis with the GMDR-GPU software, we applied it to the WTCCC dataset for the T2D phenotype [25]. Prior to analysis, we performed data quality control, checking separately for cases and control subjects, and removed those SNPs with >10% missing data or minor allele frequency of <0.05. We further performed the Hardy-Weinberg Equilibrium (HWE) test for all the SNPs included in the dataset for the control subjects and removed those SNPs with a *P* value <0.001. Following those filtering steps, 351,976 SNPs remained for each subject and were used in this study.

For the G×G analysis, we performed searches from two- to five-way interactions (Table 2). To avoid interactions that might be attributed to linkage disequilibrium effects [6], those SNP combinations containing any SNP pair whose physical distance is <1 Mb were ignored. Based on the values from 10-fold cross-validation for a given SNP combination, only those combinations whose cross-validation consistencies were >5/10 were used. An exhaustive two-dimensional search was run among those 351,976 SNPs that passed quality control testing for detecting two-way interactions.

**Table 2:** Results of two- to five-way SNP-SNP interaction analysis of WTCCC T2D data.

Characteristic	Two-way	Three-way	Four-way	Five-way
SNPs	351,976	281	281	281
Running time (hr)	31	15.2	8.2	35.9
Detected interactions	203	16	13	175
Highest testing accuracy	0.557	0.559	0.567	0.569
P value	<10^−7^	<10^−7^	<10^−7^	<10^−7^

Because it was impractical to run three- or higher-dimensional searches among these 351,976 SNPs, exhaustive three- to five-way searches were run only among the SNPs in the best combinations generated from the exhaustive two-dimensional search, a commonly used approach in G×G analysis for large dataset like GWAS [5], [6]. On the basis of the estimated time required for each run and the available computational capacity, we selected the top 1,000 SNP combinations from each training set. Because many SNPs in those best-SNP combination outputs through two-dimensional search overlapped, we finally identified 281 top and unique SNPs according to the two-dimensional search results and used them for exhaustive searches in three- to five-dimensional G×G analyses. Also, although we wished to perform as many permutations as possible for each SNP combination with our GMDR-GPU program, we finally decided to run 10^7^ permutations for each SNP combination based on our current computational capacity and the time required to complete each run. Specifically, for our report on the WTCCC T2D data, we removed those interactions whose cross-validation consistencies were <7/10 or whose *P* value was >10^−7^. After these filtering steps, the remaining best SNP combinations were considered to be significant interactions and are reported here.

### Determination of Core SNPs based on Two- to Five-way Interaction Analysis

**Figure 2:**
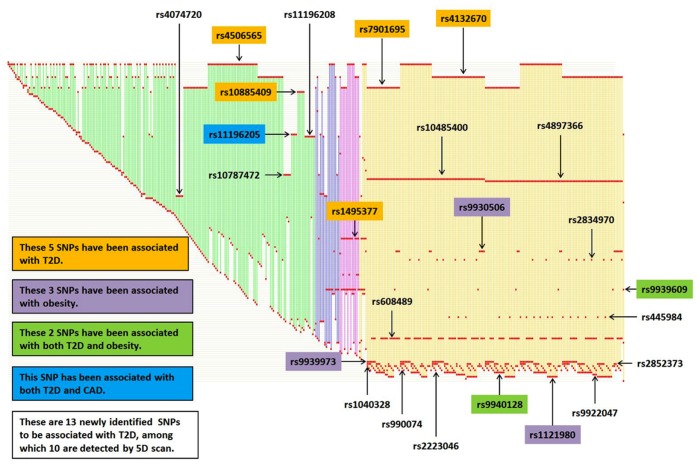
Determination of core SNPs for T2D through two-way to five-way interaction analysis using GMDR-GPU. Each SNP is represented by a red dot and each interaction by a vertical line. The red dots in the same horizontal line correspond to the same unique SNP. Different colors of vertical lines represent different interaction dimensions (green: two-way; blue: three-way; pink: four-way; yellow: five-way). All identified core SNPs are indicated with their IDs.

Following the two- to five-way interaction analyses for the T2D phenotype with GMDR-GPU, we generated an SNP-SNP interaction network with the goal of identifying core SNPs that were consistently detected by our G×G analyses at different dimensions (Figure 2). As shown in Table 3 and Figure 2, although a significant number of SNPs were identified, only 24 were considered to be “core” SNPs (i.e., identified repeatedly in multiple SNP combinations at different levels), which included 5 SNPs identified by two-way analysis; 1 by four-way analysis; 3 by two-way and five-way analyses; 1 by three-way, four-way, and five-way analyses; and the remaining 14 by five-way analysis. The best interaction models for these 24 core SNPs and their CVCs, testing accuracies, and *P* values based on 10^7^ permutated samples are shown in Table 3.

**Table 3:** Best interaction models and their cross-validation consistencies (CVC), testing accuracies, and permutated P values based on 10^7^ permutated samples for the identified 24 core SNPs.

Core SNP	Best Interaction Modelrs SNP IDs (Chrom)	CVC	Testing Accuracy	Permuted P value
rs10485400	**rs10485400**-rs4132670-rs9940128-rs608489-rs445984 (Chrom: 6-10-16-18-21)	9/10	0.568	<10^−7^
rs4897366	**rs4897366**-rs7901695-rs1121980-rs608489-rs445984 (Chrom: 6-10-16-18-21)	9/10	0.569	<10^−7^
rs4074720	rs1469244-rs2452941-**rs4074720**-rs1495377-rs9939609 (Chrom: 4-6-10-12-16)	8/10	0.565	<10^−7^
rs7901695	rs4897366-**rs7901695**-rs1121980-rs608489-rs445984 (Chrom: 6-10-16-18-21)	9/10	0.569	<10^−7^
rs4506565	rs4897366-**rs4506565**-rs1121980-rs608489-rs445984 (Chrom: 6-10-16-18-21)	9/10	0.569	<10^−7^
rs4132670	rs10485400-**rs4132670**-rs9940128-rs608489-rs445984 (Chrom: 6-10-16-18-21)	9/10	0.568	<10^−7^
rs10787472	rs2452941-**rs10787472**-rs1495377 (Chrom: 6-10-12)	7/10	0.559	<10^−7^
rs11196205	rs955436-rs6470289-**rs11196205**-rs12879941-rs9939609 (Chrom: 8-8-10-14-16)	7/10	0.565	<10^−7^
rs10885409	rs12154976-**rs10885409**-rs2457179 (Chrom: 7-10-11)	8/10	0.557	<10^−7^
rs11196208	rs17608635-rs2736010-**rs11196208**-rs12879941-rs9939609 (Chrom: 4-8-10-14-16)	7/10	0.568	<10^−7^
rs1495377	rs4708273-rs4506565-**rs1495377**-rs9930506-rs11665417 (Chrom: 6-10-12-16-18)	7/10	0.567	<10^−7^
rs9939973	rs4897366-rs4506565-**rs9939973**-rs2852373-rs445984 (Chrom: 6-10-16-18-21)	10/10	0.568	<10^−7^
rs9940128	rs4897366-rs7901695-**rs9940128**-rs608489-rs445984 (Chrom: 6-10-16-18-21)	9/10	0.568	<10^−7^
rs9922047	rs10485400-rs4132670-**rs9922047**-rs608489-rs2834970 (Chrom: 6-10-16-18-21)	7/10	0.565	<10^−7^
rs1121980	rs4897366-rs7901695-**rs1121980**-rs608489-rs445984 (Chrom: 6-10-16-18-21)	9/10	0.569	<10^−7^
rs9939609	rs17608635-rs2736010-rs11196208-rs12879941-**rs9939609** (Chrom: 4-8-10-14-16)	7/10	0.568	<10^−7^
rs9930506	rs10485400-rs4132670-**rs9930506**-rs608489-rs2223046 (Chrom: 6-10-16-18-21)	8/10	0.568	<10^−7^
rs2852373	rs4897366-rs4506565-rs1121980-**rs2852373**-rs445984 (Chrom: 6-10-16-18-21)	8/10	0.569	<10^−7^
rs608489	rs4897366-rs7901695-rs1121980-**rs608489**-rs445984 (Chrom: 6-10-16-18-21)	9/10	0.569	<10^−7^
rs445984	rs4897366-rs7901695-rs1121980-rs608489-**rs445984** (Chrom: 6-10-16-18-21)	9/10	0.569	<10^−7^
rs1040328	rs10485400-rs7901695-rs9930506-rs608489-**rs1040328** (Chrom: 6-10-16-18-21)	8/10	0.567	<10^−7^
rs990074	rs10485400-rs7901695-rs9930506-rs608489-**rs990074** (Chrom: 6-10-16-18-21)	7/10	0.565	<10^−7^
rs2223046	rs10485400-rs4132670-rs9930506-rs608489-**rs2223046** (Chrom: 6-10-16-18-21)	8/10	0.568	<10^−7^
rs2834970	rs10485400-rs4132670-rs9922047-rs608489-**rs2834970** (Chrom: 6-10-16-18-21)	7/10	0.565	<10^−7^

Note: In our GMDR-GPU analysis, age and sex were used as covariates, and BMI was not adjusted for.

Further mapping and bioinformatics analysis of the 24 core SNPs revealed that they are located in six genes (Table 4), with 2 SNPs in the l(3)mbt-like 3 gene (*L3MBTL3*) on chromosome 6; 8 in the transcription factor 7-like 2 gene (*TCF7L2*) on chromosome 10; 1 in the tetraspanin 8 gene (*TSPAN8*) on chromosome 12; 6 in the fat mass and obesity-associated gene (*FTO*) on chromosome 16; 2 in the CUGBP, Elav-like family member 4 gene (*CELF4*) on chromosome 16; and 5 in the runt-related transcription factor 1 gene (*RUNX1*) on chromosome 21. Of these six genes, *TCF7L2*, *TSPAN8*, and *FTO* have been previously reported to be associated with T2D [25], [27]–[31], whereas other three genes (i.e., *L3MBTL3*, *CELF4*, and *RUNX1*) have not; thus, they likely represent new susceptibility genes for T2D in WTCCC GWAS dataset.

**Table 4:** Core SNPs associated with T2D based on G×G analysis at different orders with GMDR-GPU program.

Core SNP ID (Alleles)	Gene and Location^1^	Physical Location^2^	Coding	Chrom	RefSeq	Reported Disease & Reference
rs10485400 (A/G)	*L3MBTL3* (l(3)mbt-like 3; Drosophila)	130485817	Intron	6	NC_000006.10	
rs4897366 (A/C)		130499771	Intron			
rs4074720 (A/G)		114738487	Intron			
rs7901695 (C/T)	*TCF7L2*	114744078	Intron			T2D [34]
rs4506565 (A/T)	(transcription	114746031	Intron			T2D [28]
rs4132670 (C/T)	factor 7-like 2;	114757761	Intron	10	NC_000010.9	T2D [35]
rs10787472 (A/C)	T-cell specific,	114771287	Intron			
rs11196205 (C/G)	HMG-box)	114797037	Intron			T2D [27], CAD [36]
rs10885409 (C/T)		114798062	Intron			T2D [33]
rs11196208 (C/T)		114801306	Intron			
rs1495377 (C/G)	*TSPAN8* (tetraspanin 8)	69863368	Intron	12	NC_000012.10	T2D [25]
rs9939973 (A/G)		52358069	Intron			Obesity [37]
rs9940128 (A/G)	*FTO* (fat mass	52358255	Intron			T2D [40], Obesity [41]
rs9922047 (C/G)	and obesity	52363781	Intron	16	NC_000016.8	
rs1121980 (C/T)	associated)	52366748	Intron			Obesity [38]
rs9939609 (A/T)		52378028	Intron			T2D [43], Obesity [42]
rs9930506 (A/G)		52387966	Intron			Obesity [39]
rs2852373 (C/T)	*CELF4* (CUGBP, Elav-like family member 4)	33157857	Intron	18	NC_000018.8	
rs608489 (A/G)		33159109	Intron			
rs445984 (C/T)		35819728	Intron			
rs1040328 (C/T)	*RUNX1* (runt-	35832534	Intron			
rs990074 (C/T)	related	35833022	Intron	21	NC_000021.7	
rs2223046 (A/G)	transcription	35833974	Intron			
rs2834970 (A/G)	factor 1)	35847430	Intron			

1 Gene locations were retrieved from Enzembl Genome Browser.

2 Physical location of each gene determined according to NCBI build 36.

## Discussion

As more and more GWAS studies are conducted throughout the world, efficient methodologies and computer programs for detecting G×G in the data have become essential and a challenge for many researchers [6], [7], [19]. To meet this challenge, we developed a new GPU-based software, called GMDR-GPU, which represents a significant extension of our previously reported GMDR [17]. With this newly developed software, it becomes possible to search for G×G in GWAS data within a reasonable time. However, there still exist several potential limitations on the current version of the program with most of them more or less related to the computational capability available to us rather than to the program per se. For example, we are not sure whether our program could perform G×G analysis in a GWAS dataset containing more than 10,000 subjects, as we did not have access to any GWAS dataset with such large sample. In our experience, although it is possible, the likelihood of any single GWAS dataset having a sample size of more than 10,000 is very low or close to zero except for a combined GWAS dataset from different projects where various meta-analysis approaches should be used to merge G×G results across different datasets as we commonly did for the single-locus GWAS analysis. As stated earlier, we could produce only a precise p value of 10^−7^ because our available computational capacity can perform only 10^7^ permutations within a reasonable time. We acknowledge that using a p value of 10^−7^ as the threshold for pair-wise G×G analysis in a GWAS dataset is not as stringent as we wish for this type of analysis. However, it is our believe that such a threshold could easily be changed with the improvement of computational capacity. Finally, we performed G×G analysis only for all possible pair-wise SNP combinations but not for higher-order G×G analysis because of our limited computational capability now. Even though there existed the abovementioned limitations in our current study, our newly developed GMDR-GPU still represents one of the most advanced tools available in the field for performing G×G analysis in a GWAS dataset.

As a demonstration of the GMDR-GPU program, we used it to analyze the WTCCC GWAS data for T2D phenotype. Through a series of analytical approaches, including G×G analysis at different dimensions, core SNP detection, and network analysis, we identified six susceptibility genes for T2D in the WTCCC dataset. Of these genes, there is convincing evidence supporting the involvement of variants in three, namely *TCF7L2* [27], [28], *TSPAN8* [25], [29], and *FTO* [30]–[32], in T2D. For example, four SNPs (*i.e*., rs4506565 [28], rs10885409 [33], rs7901695 [34], and rs4132670 [35]) in *TCF7L2* have been reported to be associated with T2D, and SNP rs11196205 in *TCF7L2* is associated with both T2D and coronary artery disease [27], [36]. Here, we discovered that three more SNPs (i.e., rs4074720, rs10787472, and rs11196208) in the same genes are involved in the etiology of T2D through G×G. For the *TSPAN8* gene, SNP rs1495377 has been reported to be associated with T2D [25]. For *FTO*, three SNPs (rs9939973 [37], rs1121980 [38], and rs9930506 [39]) have been reported to be associated with obesity and two SNPs (rs9940128 [40], [41] and rs9939609 [42], [43]) with both T2D and obesity. We found one new SNP (i.e., rs9922047) in *FTO* to be associated with T2D.

Importantly, we identified three novel susceptibility genes for T2D (i.e., *L3MBTL3* on chromosome 6, *CELF4* on chromosome 18, and *RUNX1* on chromosome 21), with two SNPs in *L3MBTL3* (rs10485400 and rs4897366), two in *CELF4* (rs2852373 and rs608489), and five in *RUNX1* (rs445984, rs1040328, rs990074, rs2223046 and rs2834970). Whether the three genes are indeed involved in T2D and how they are involved awaits further replication in independent studies. Although these three genes have not been reported to be associated with T2D, they have been thought to be involved in other human diseases or complex traits. For example, rs6899976 in *L3MBTL3* is associated with human adult height [44] and *rs6569648* with birth height [45]. *CELF4* is a family member of structurally related RNA-binding proteins involved in various aspects of RNA processing, including splicing and mRNA stability [46], and is expressed widely during development but is restricted to the central nervous system in adults [47]. Increasing evidence has implicated the involvement of CELF proteins in various neurologic disorders such as type 1 myotonic dystrophy, spinal muscular atrophy, and seizures and epilepsy [46]. Although a link of T2D with RNA-binding proteins awaits further verification with independent studies, this could be significant if it proves to be true because it implies that RNA processing plays an important role in the pathology of T2D. Genetic variations in *RUNX1* are associated with colon and rectal cancer [48].

**Figure 3:**
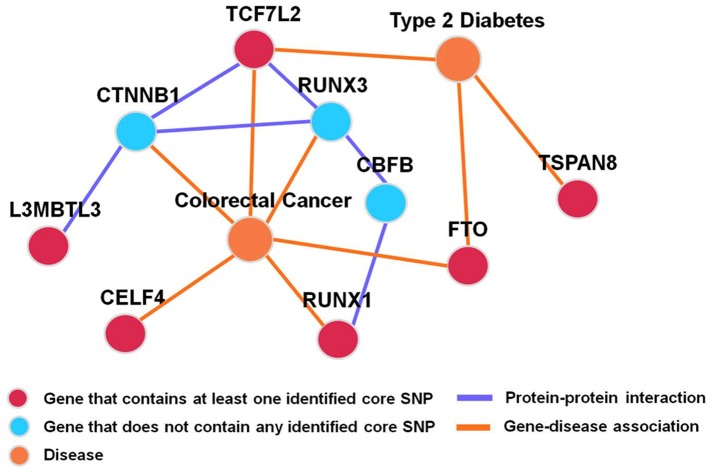
Interaction/association network among the six genes containing at least one core SNPs identified by analyzing WTCCC T2D data with GMDR-GPU. Beyond the six susceptibility genes identified in this work, three other genes, *CTNNB1*, *RUNX3*, and *CBFB*, were found by a literature search. Although these genes have been associated with a number of human disorders, only two closely related diseases, i.e., T2D and colorectal cancer, are shown.

Our gene network investigation provides more evidence for the involvement of the identified six genes in T2D (see Figure 3). As described above, genes *TCF7L2*, *TSPAN8*, and *FTO* have previously been associated with T2D; and our current study provided further support for their involvement. For *L3MBTL3* and *RUNX1*, although we failed to identify any report of their involvement in T2D, they have been shown to interact with *TCF7L2* indirectly at the protein level. For example, *L3MBTL3* interacts with the catenin (cadherin-associated protein) beta 1 gene (*CTNNB1*) [49], while *CTNNB1* interacts with *TCF7L2* [50]. *RUNX1* interacts with the core-binding factor beta subunit gene (*CBFB*) [51], which interacts with *RUNX3* [51], while *RUNX3* interacts with *TCF7L2* [52]. Interestingly, *RUNX3* also interacts with *CTNNB1* [52]. Finally, it is worth pointing out that *CELF4* [53], *TCF7L2* [54], *FTO* [55], *RUNX1* [48], *RUNX3* [48], and *CTNNB1* [56] have all been reported to be involved in the etiology of colorectal cancer. This makes our findings more convincing and attractive because it is well documented that T2D is associated with an increased risk of colorectal cancer [57], [58]. Regarding the results of our G×G analysis on WTCCC T2D data, we point out that it would be far more convincing if we could replicate these findings in other independent samples. However, given that the primary goal of this communication is to report the development of the GMDR-GPU program such that other researchers can begin to use it to analyze their datasets, we consider validation of these newly identified loci for T2D and further analysis of the dataset for other phenotypes including T2D as logical steps for future research, which are beyond the scope of this paper.

Although MDR or its derivatives have identified numerous G×G variants for many human diseases at the individual gene level [19], there exist some significant limitations of these approaches. The first lies in the computing program codes themselves, which are incapable of handling GWAS data, as they were not designed with genome-wide data in mind [6]. Another potential limitation of the MDR software or its derivatives is that it produces only what is considered to be the “best” interaction model rather than multiple models with similar statistical characteristics. To overcome these limitations and meet the demands of human genetics researchers, we implemented our original GMDR algorithm [17] on a computing system with GPUs, a type of hardware implementation of parallel computation that can be adapted to many scientific tasks. Because of its many-core architecture, a significant number of threads can be run simultaneously so that massively parallel computation can be performed in a more cost-effective way, even on personal computers. For example, if one wants to reach the same speed of GMDR-GPU with CPU versions, one would need to build a cluster consisting of at least 550 CPUs, which is much more expensive and consumes much more power than a Tesla C2070 GPU. Further, it probably is impractical to apply the original Java implementation to an exhaustive search among millions of SNPs, because this requires a computer equipped with thousands of CPUs to reach the same speed in order to handle such a dataset with a month.

In sum, by taking advantage of the massively parallel computing technology of GPU, our newly developed GMDR-GPU software is able to overcome the computational bottleneck of the original GMDR software and perform exhaustive searches of G×G on GWAS data. Following the development of GPU-based GMDR-GPU program, we analyzed WTCCC dataset for T2D phenotype and our obtained results not only confirmed some earlier findings (such as the well-documented associations of *TCF7L2*, *TSPAN8*, and *FTO* variants with T2D) but also identified three more susceptibility genes (i.e., *L3MBTL3*, *CELF4*, and *RUNX1*) that have not been associated with T2D before. Since the association of the three genes with T2D has not been replicated in independent samples, we caution that these findings are tentative, and replication studies are warranted.
